# The Impact of Increased Bladder Blood Flow on Storage Symptoms after Holmium Laser Enucleation of the Prostate

**DOI:** 10.1371/journal.pone.0129111

**Published:** 2015-06-19

**Authors:** Keisuke Saito, Shin-ichi Hisasue, Hisamitsu Ide, Hiroaki Aoki, Satoru Muto, Raizo Yamaguchi, Akira Tsujimura, Shigeo Horie

**Affiliations:** 1 Department of Urology, Juntendo University, Graduate School of Medicine, Hongo 2-1-1, Bunkyo-ku, Tokyo, 113–0033, Japan; 2 Department of Urology, Teikyo University, School of Medicine, Kaga 2-11-1, Itabashi-ku, Tokyo, 173–8605, Japan; King's College London, UNITED KINGDOM

## Abstract

In order to investigate how holmium laser enucleation of the prostate (HoLEP) improves urinary storage symptoms, we assessed blood flow in the urinary bladder mucosa of patients with benign prostatic hyperplasia (BPH) before and after laser surgery. Seventy-four consecutive patients with BPH (median age 69 years, range; 53–88) underwent HoLEP at our institution and are included in this study. We prospectively assessed the International Prostate Symptom Score (IPSS), IPSS-QOL Score, the Overactive Bladder Symptom Score (OABSS), uroflowmetry, and blood flow in the urinary bladder, before and after surgery. Blood flow in the bladder mucosa was measured using the OMEGA FLOW (OMEGAWAVE, Tokyo, Japan) laser Doppler flowmeter. The median volume of the enucleated adenomas was 45.0 g (range: 25.0 to 83.2). The median IPSS improved significantly from 20 (range: 6–35) to 3 (0–22) (p<0.001; Wilcoxon signed-rank test), as did the storage symptoms score, which decreased from 13 (2–20) to 3 (1–8) (p<0.001). Median bladder blood flow increased at the trigone from 9.57±0.83 ml/sec to 17.60±1.08 ml/sec. Multiple regression analysis for the improved storage symptom score eliminated all explanatory variables except increased bladder perfusion. The data suggest that HoLEP improves blood flow in the bladder mucosa, which independently leads to the improvement of storage symptoms.

## Introduction

Holmium laser enucleation of the prostate (HoLEP) has become a gold standard treatment for prostate enlargement.[1] The expanding adenoma in benign prostatic hyperplasia (BPH) creates a natural tissue plane that can be exploited by HoLEP without destroying the bladder neck. HoLEP enables the immediate relief of bladder outlet obstruction and produces an excellent outcome in terms of feasibility, safety, and efficacy.[1, 2] Storage symptoms are currently largely encompassed by the term "overactive bladder syndrome" or OABS. The symptoms produced by OAB are believed to correlate with underlying overactivity of the detrusor muscle.[3] Women with OAB experience identical symptoms to men, but the symptoms originate primarily from the bladder.[4] In men, particularly older men with BPH, symptoms of OAB arise from secondary causes, including prostatic pathology. Several reports have shown relief of OAB symptoms in men following surgery for BPH.[5, 6]

The mechanism that underlies the improvement of storage symptoms observed following surgery remains unclear. Recently, however, several new lines of evidence suggest that chronic ischemia of the urinary bladder is associated with, and therefore, may cause lower urinary tract symptoms (LUTS).[7, 8] To examine this hypothesis, we used endoscopic laser Doppler flowmeter (LDF), a non-invasive method that enables monitoring of microvascular blood flow, to compare blood perfusion in bladders of patients with BPH before and after HoLEP to assess the efficacy of HoLEP in mitigating lower urinary tract symptoms.

## Materials and Methods

### Patients

Patients were enrolled in this study with the approval of the institutional review board of Teikyo University Hospital. Written, informed consent was obtained from all participants. A complete medical history was taken and physical examination was performed including digital rectal examination, urinalysis, serum prostate-specific antigen (PSA) level, transabdominal ultrasonography, uroflowmetry, and postvoid residual urine volume. Before HoLEP, 1 week after, and every 3 months thereafter, the International Prostate Symptom Score (IPSS), the Overactive Bladder Symptom Score (OABSS), uroflowmetry, and postvoid residual urine volume obtained by ultrasonography were measured. A prostate biopsy was performed before HoLEP, when the serum PSA level was elevated (>2.5 ng/ml), or when the digital rectal examination was abnormal. Patients with prostate cancer or a history of previous prostate surgery were excluded from this study. The current study was approved by the Teikyo University Institutional Review Board (No. 09–095). All participants provided written informed consent to participate in this study. The Institutional Review Board also approved the consent procedures.

### HoLEP

A 26-Fr. Storz endoscope (KARL STORZ INC., Tuttlingen, Germany) with a continuous saline irrigation system equipped with a device for fixing the 550-μm-laser fiber was used. A pulsed, high-powered holmium neodymium: yttrium-aluminum garnet laser was generated by Versa Pulse Select 80 (LUMENIS INC., CA, U.S.A). Transurethral mechanical morcellation was performed. Most of the procedures (68 of 74: 91.9%) were performed by one surgeon (K.S.).

### Measurement of the surface blood flow of the bladder by a laser Doppler flowmeter

**Fig 1:**
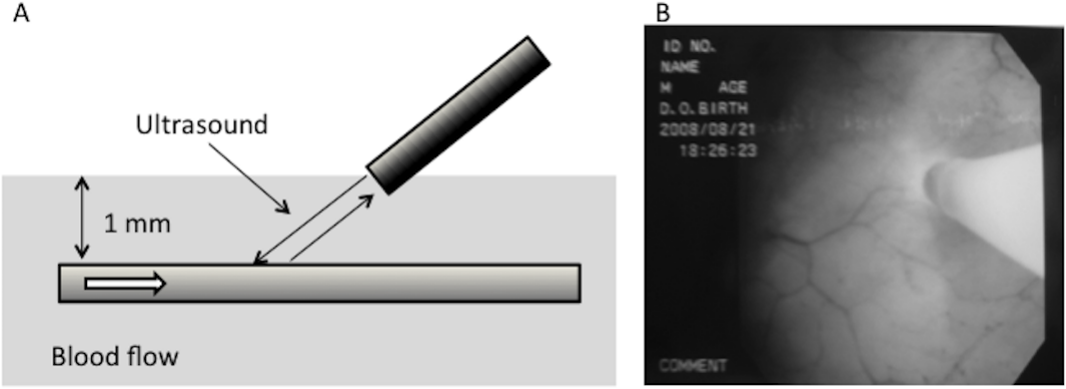
Laser Doppler flowmeter (LDF; OMEGA FLOW FLO-C1 BV, OMEGAWAVE, Tokyo, Japan). 1A: LDF employs a beam of low intensity monochromatic infrared light emitted from a laser diode within the flowmeter to measure the real-time micro-perfusion rate at tissue depths of 1 mm. B: The probe is placed a few mm away from the bladder mucosa while the bladder is filled with 150 ml saline at room temperature.

The Laser Doppler Flowmeter (LDF; OMEGA FLOW FLO-C1 BV, OMEGAWAVE, Tokyo, Japan) was used to measure surface blood flow in the bladder; once before HoLEP and once in the interval between 3 and 6 months after HoLEP. The LDF uses a beam of low intensity monochromatic infrared light that is emitted from a laser diode within the flowmeter. The diode is able to measure the real-time micro-perfusion rate in tissue at a depth of 1 mm (Fig 1A). The beam travels through a 0.5 mm semiconductor laser probe in a cystoscope, the end of which illuminates the tissue under study. A fraction of the light beam is reflected by the moving red blood cells within the tissue and undergoes a frequency shift due to Doppler effect that is proportional to the average red cell velocity. The reflected signal is processed in real time to give a measurement of tissue perfusion. The probe was placed a few mm away from the bladder mucosa of the trigone while the bladder was filled with 150 ml of saline at room temperature (Fig 1B). A laser probe in the cystoscope measures the micro-perfusion of the bladder mucosa without touching the mucosa. Measurements were taken of the trigone as well as of the left and right lateral wall of the bladder.

### Statistical analysis

The data were analyzed by the Wilcoxon Signed rank-test, Dunnet’s test, Student’s t-test and presented as median (range) and mean ± the standard error of the mean (SEM). Multiple regression analysis was performed to develop a model for predicting improvement of storage symptoms. The regression models were constructed with age, enucleated prostate volume, increased bladder perfusion rate, change of postvoid residual, and baseline OABSS. For all statistical comparisons, significance was defined as p<0.05.

## Results

Seventy-four consecutive patients (median age 69 years, range; 53–88) who underwent HoLEP at our institution were included in this study. The clinical characteristics of this patient group are shown in Table 1. None of the patients required blood transfusions, and no hyponatremia was found in any patient during or after the operation.

**Table 1:** Patient characteristics.

Variables	Median (range)
Age (years)	69 (53–88)
PSA (ng/ml)	10.2 (0.2–69.6)
TZ volume (cm^2^)	54.4 (21.5–162.0)
Enucleated adenoma weight (g)	45.0 (25.0–83.2)
IPSS	20 (6–35)
QOL	5 (0–13)
OABSS	6 (0–13)

Abbreviations: PSA is prostate-specific antigen; TZ is transition zone; IPSS is International Prostate Symptom Score; QOL is Quality of Life score: OABSS is Overactive Bladder Symptom Score

**Fig 2:**
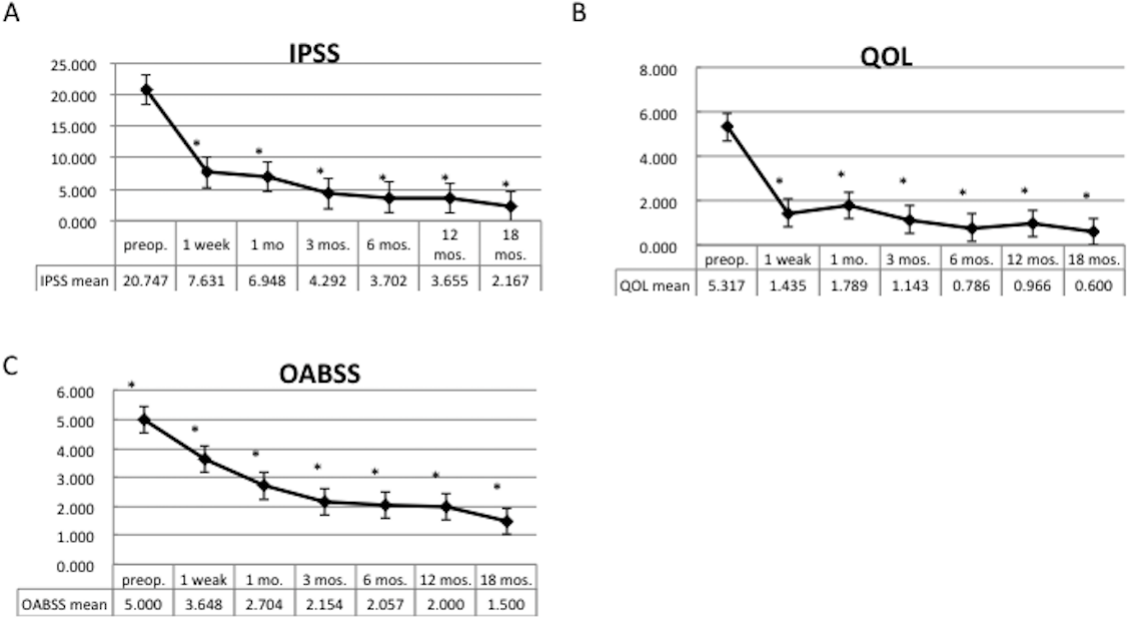
Longitudinal change in IPSS (A), QOL score (B), and OABSS (C). There was significant improvement 1 week after surgery (*: p<0.05 Dunnett's test, compared with baseline). Note that the improvement of the OABSS is slower than the IPSS score.

**Fig 3:**
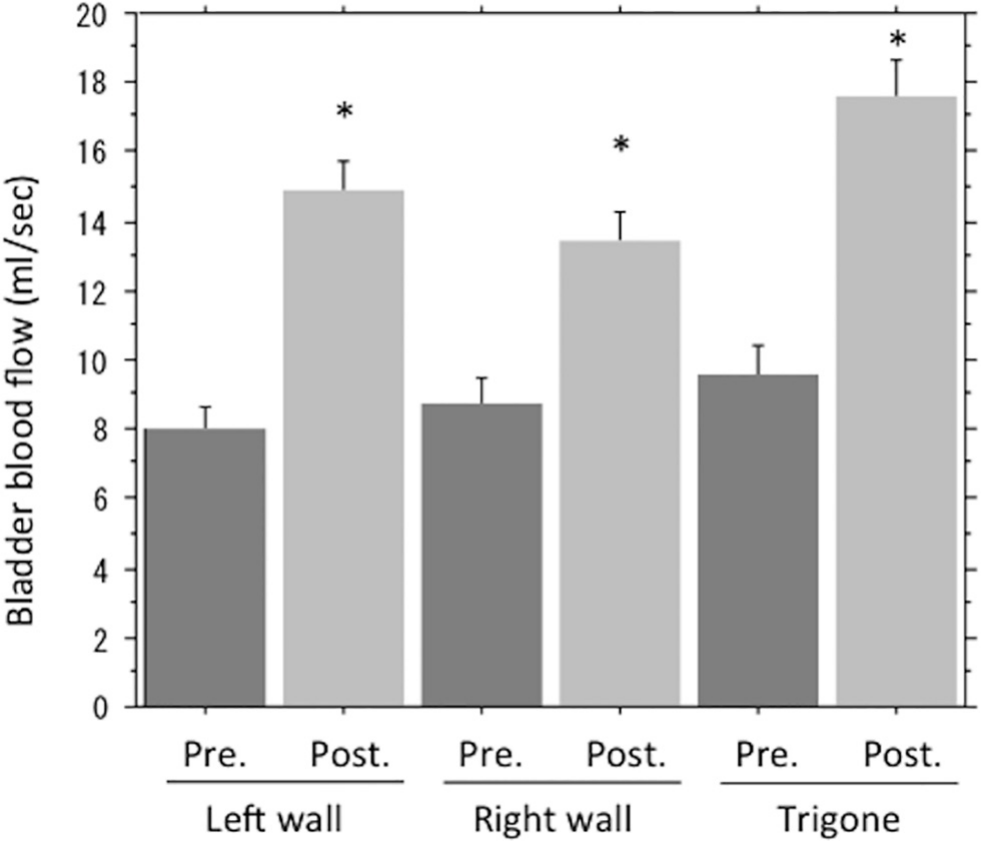
Bladder blood flow measured by LDF. Every region of the bladder including left wall, right wall, and bladder trigone showed a significant increase in blood flow as measured by LDF. *: p<0.001 Student’s t-test

Table 2 shows the results of clinical assessment performed 6 months after HoLEP. Subjective symptoms, including IPSS, QOL score, and OABSS, all improved significantly from baseline after HoLEP. Longitudinal outcomes of IPSS, QOL scores, and OABSS are shown in Fig 2A, 2B and 2C. Objective parameters were also significantly improved as evidenced by the maximum flow rate, voided volume, and postvoid residual urine volume after surgery. The surface blood flow increased significantly after surgery in all regions of the bladder. Specifically, surface blood flow increased from 8.07±0.62 ml/sec to 14.90±0.86 ml/sec in the left wall, from 8.78±0.74 ml/sec to 13.40±0.85 ml/sec in the right wall, and from 9.57±0.83 ml/sec to 17.60±1.08 ml/sec at the trigone (mean±SEM). (Fig 3) The measured surface blood flow in the bladder trigone was used for the further analysis.

**Table 2:** Change in individual parameters before and after HoLEP.

Variables	Before HoLEP	After HoLEP (6 months)	P-value
IPSS	20 (6–35)	3 (0–22)	< 0.001
Voiding symptom	9 (0–15)	0 (0–4)	< 0.001
Storage symptom	13 (2–10)	3 (1–8)	< 0.001
QOL	6 (2–13)	1 (0–5)	< 0.001
OABSS	5 (0–13)	2 (0–7)	< 0.001
Voided Volume (ml)	94 (0–434)	148 (0–505)	0.007
Max flow rate	6.55(2.4–15.7)	16.6(3.8–40.9)	< 0.001
Postvoid residual (ml)	85.5(0–999)	24.0(0–124)	< 0.001

Data are derived from the Wilcoxon’s signed rank test

**Fig 4:**
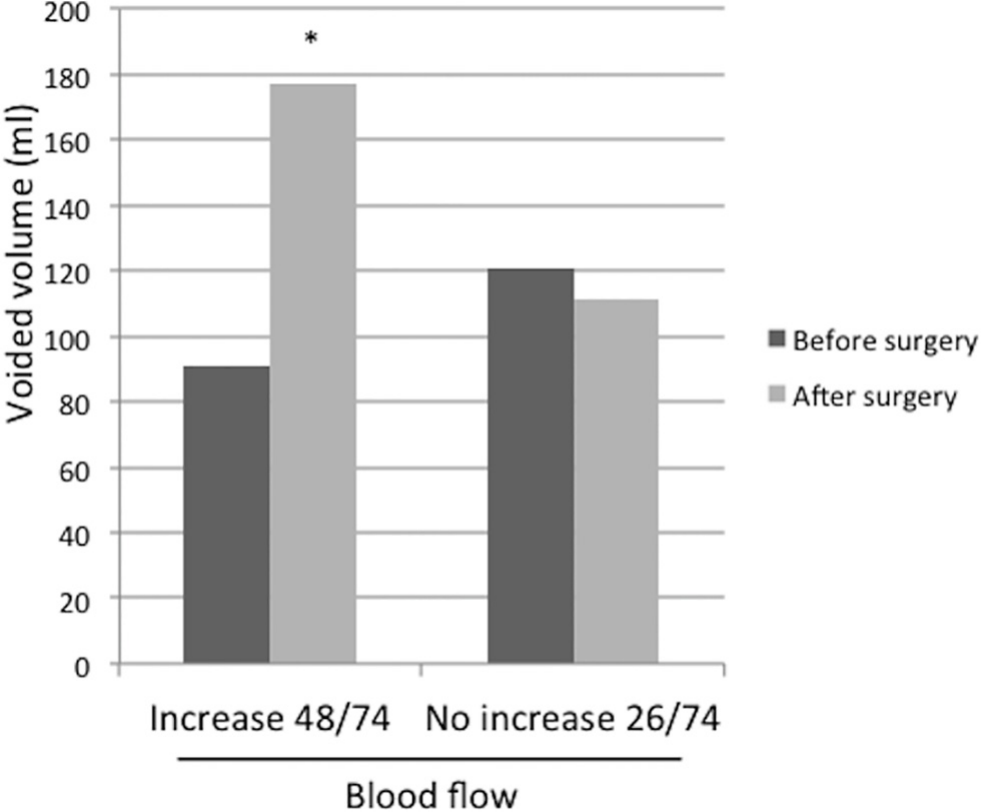
The change in voided volume. The change in voided volume is shown before and after surgery for both the increased perfusion group and the no change group. *: p<0.0001 Student’s t-test

**Fig 5:**
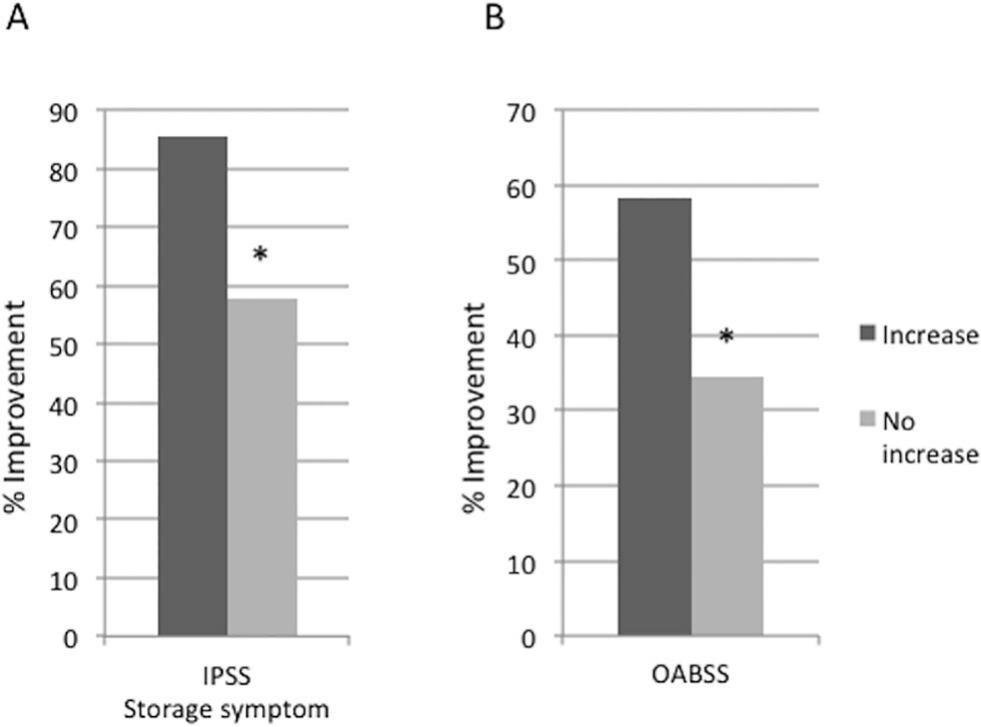
The improvement rate of IPSS and OABSS. (A) shows the improvement rate of storage symptoms in IPSS for both the increased perfusion group and the no change group. (B) shows the improvement rate of OABSS storage symptoms for the increased perfusion group and the no change group.*: p<0.001 Student’s t-test

Multiple regression analysis eliminated all explanatory variables except for the effect of increased bladder perfusion on storage symptoms (Table 3). In 48 of 74 cases, bladder perfusion was significantly increased (7.64 vs. 20.01 ml/sec at the bladder trigone) based on measurements taken before and after HoLEP (p<0.001). The change was less than 5% in the remaining 26 patients (12.30 vs. 12.64, p = 0.495). In the group with increased blood flow after HoLEP, the voided volume measured by uroflowmetry was significantly increased (91 vs. 177.5, p<0.0001, Fig 4), and the improvement of storage symptom scores in both the IPSS and OABSS was also significant (IPSS 85.4% vs. 57.7%, p<0.0001, OABSS 58.3% vs. 34.6%, p<0.0001, Fig 5A and 5B). However, for the group that did not show an increase of bladder blood flow, there was no change in voided volume, storage symptoms, or OABSS.

**Table 3:** Multiple regression analysis for the improvement of the storage symptoms.

	Unstandardized Coefficients B	SE	Standardized Coefficients Beta	t	p-value
Age	-0.301	0.450	-0.119	-0.669	0.509
Enucleated prostate volume	-0.029	0.077	-0.068	-0.378	0.708
Increased bladder perfusion rate	0.480	0.229	0.374	2.102	0.044
Change of postvoid residual	0.002	0.019	0.022	0.129	0.899
Baseline OABSS	-0.288	0.846	-0.062	-0.340	0.736

## Discussion

The current study sought to determine the efficacy of HoLEP on urinary symptoms using the endoscopic laser Doppler flowmeter (LDF) to compare blood perfusion in the bladders of patients with BPH before and after HoLEP. In addition to relief of voiding symptoms, our findings show that HoLEP significantly reduced the severity of storage symptoms in patients undergoing this procedure. Overactivity of detrusor muscle has been identified in approximately 45%–50% of men with bladder outlet obstruction and it has been postulated that detrusor overactivity exacerbates storage symptoms in BPH. The mechanisms thought to be related to this phenomenon are denervation hypersensitivity of the cholinergic receptors (Cannon’s law) in detrusor muscle and/or structural changes resulting from urinary bladder ischemia. [9] Histological changes in bladder tissue, along with fibrosis and bladder dysfunction, are induced by atherosclerosis in rabbits, and moderate bladder ischemia has been shown to increase detrusor muscle overactivity.[8] Thus, bladder ischemia appears to play a key role in detrusor overactivity.

In this study, multivariate linear regression analysis demonstrated increased blood perfusion of the bladder to be the only clinical parameter associated with improved storage symptoms. Perfusion of the bladder neck and prostate is decreased in patients with lower urinary tract symptoms examined on transrectal color Doppler ultrasonography.[10] In patients with BPH there is often continuous over-distention of the bladder due to incomplete voiding. This sustained increase in residual urine results in decreased bladder blood flow. While all of the blood vessels are open at 5% bladder capacity; most veins are collapsed at 25% bladder capacity, and a greater percentage of vessels become collapsed with increasing bladder volumes.[11] Furthermore, in the rabbit model, partial outlet obstruction reduced vesical blood flow, which in turn induced degenerative changes in the bladder.[7] It has been suggested the chronic ischemia of the bladder associated with detrusor overactivity is the result of histological disruption of the urothelium.[8] These studies appear to indicate that chronic bladder ischemia is associated with irritative and/or storage symptoms of the lower urinary tract in BPH.

It remains less well studied whether surgical treatment of BPH is associated with increased bladder blood flow. One report has indicated that transurethral resection of the prostate (TURP) improves urinary symptoms accompanied by a decreased incidence of detrusor instability from 60% to 25%.[5] Another study reported that patients who exhibited continued detrusor overactivity after TURP, did not have increased bladder perfusion on color Doppler transrectal ultrasonography.[12] Yet another study using contrast-enhanced color Doppler ultrasonography assessed bladder vascular resistance, using the resistive index (RI) of vesical arteries. The preoperative RI was significantly higher in patients with enlarged prostates (> 60 ml) and severe obstruction. Furthermore, RI decreased significantly after TURP. In our study, we utilized laser Doppler flowmeter to get a more precise assessment of bladder perfusion, and the findings clearly show that the surface blood flow of the bladder mucosa is significantly increased after HoLEP, concomitant with an improvement of storage symptoms. Remodeling blood perfusion in the bladder therefore is considered to be key to improved storage symptoms after HoLEP.

There are several limitations in this study. We did not assess urodynamics including pressure flow studies to assess detrusor overactivity and obstruction before and after surgery. The objective functional outcome following HoLEP and its association with bladder blood perfusion could be important factors for the mechanism of the relief of storage symptoms. Moreover, we did not assess urinalysis during follow-ups. Inflammation following surgery might be responsible for the increased bladder blood perfusion. Therefore, we need further assessment on the pressure flow urodynamic study and the inflammation following HoLEP in order to understand their relationships with bladder blood perfusion in future.

## Conclusions

Holmium laser enucleation of the prostate increases blood perfusion in the urinary bladder in most patients who undergo treatment, as measured by laser Doppler flowmeter before and after surgery. All patients with increased bladder blood flow also exhibited improvement of their lower urinary tract symptoms, including both voiding symptoms and storage symptoms. However, the only independent variable shown to be associated with increased bladder blood flow on multiple regression analysis was improved storage symptoms. This correlation between bladder blood flow and urinary symptoms suggests that bladder ischemia contributes to the exacerbation of storage symptoms in patients with BPH.
